# Paying for the Orphan Drug System: break or bend? Is it time for a new evaluation system for payers in Europe to take account of new rare disease treatments?

**DOI:** 10.1186/1750-1172-7-74

**Published:** 2012-09-26

**Authors:** Wills Hughes-Wilson, Ana Palma, Ad Schuurman, Steven Simoens

**Affiliations:** 1 EU Committee of Experts on Rare Diseases (EUCERD), Global Public Policy & Government Relations, SOBI – Swedish Orphan Biovitrum, Tomtebodvägen, 23a, Solna, Sweden; 2 Market Access & Public Affairs, Shire Human Genetic Therapies, Lambroekstraat 5C, B-1831, Diegem, Belgium; 3 Business Contact Centre & International Affairs, Dutch Health-Care Insurance Board (CVZ – College voor zorgverzekeringen), Eekholt 4, 1112 XH, Diemen, Netherlands; 4 Pharmaco-economics Katholieke Universiteit Leuven, Research Centre for Pharmaceutical Care & Pharmaco-economics O&N2 Bus 521, Herestraat 49, B-3000, Leuven, Belgium

## Abstract

Since its enactment in 2000, the European Orphan Medicinal Products Regulation has allowed the review and approval of approaching 70 treatments for some 55 different conditions in Europe. Success does not come without a price, however. Many of these so-called “orphan drugs” have higher price points than treatments for more common diseases. This has been raising debate as to whether the treatments are worth it, which, in turn risks blocking patient access to treatment. To date, orphan drugs have only accounted for a small percentage of the overall drug budget. It would appear that, with increasing numbers of orphan drugs, governments are concerned about the future budget impact and their cost-effectiveness in comparison with other healthcare interventions. Orphan drugs are under the spotlight, something that is likely to continue as the economic crisis in Europe takes hold and governments respond with austerity measures that include cuts to healthcare expenditures. Formally and informally, governments are looking at how they are going to handle orphan drugs in the future. Collaborative proposals between EU governments to better understand the value of orphan drugs are under consideration. In recent years there has been increasing criticism of behaviours in the orphan drug field, mainly centring on two key perceptions of the system: the high prices of orphan drugs and their inability to meet standard cost-effectiveness thresholds; and the construct of the system itself, which allows companies to gain the benefits that accrue from being badged as an orphan drug. The authors hypothesise that, by examining these criticisms individually, one might be able to turn these different “behaviours” into criteria for the creation of a system to evaluate new orphan drugs coming onto the market. It has been acknowledged that standard methodologies for Health Technology Assessments (HTA) will need to be tailored to take into account the specificities of orphan drugs given that the higher price-points claimed by orphan drugs are unlikely to meet current cost-effectiveness thresholds. The authors propose the development of a new assessment system based on several evaluation criteria, which would serve as a tool for Member State governments to evaluate each new orphan drug at the time of pricing and reimbursement. These should include rarity, disease severity, the availability of other alternatives (level of unmet medical need), the level of impact on the condition that the new treatment offers, whether the product can be used in one or more indications, the level of research undertaken by the developer, together with other factors, such as manufacturing complexity and follow-up measures required by regulatory or other authorities. This will allow governments to value an orphan drug that fulfilled all the criteria very differently from one that only met some of them. An individual country could determine the (monetary) value that it places on each of the different criteria, according to societal preferences, the national healthcare system and the resources at its disposal – each individual government deciding on the weighting attributed to each of the criteria in question, based on what each individual society values most. Such a systematic and transparent system will help frame a more structured dialogue between manufacturers and payers, with the involvement of the treating physicians and the patients; and foster a more certain environment to stimulate continued investment in the field. A new approach could also offer pricing and reimbursement decision-makers a tool to handle the different characteristics amongst new orphan drugs and to redistribute the national budgets in accordance with the outcome of a differentiated assessment. The authors believe that this could, therefore, facilitate the approach for all stakeholders.

## Article

Since its enactment in 2000, the European Orphan Medicinal Products Regulation [1] has allowed the review and approval of approaching 70 treatments for some 55 different conditions in Europe [2]. This compares with just eight treatments for rare and serious conditions being approved prior to the creation of the Regulation [3]. The Regulation can, therefore, be considered a success. Stakeholders in Europe, including patients and industry, have also hailed the EU Orphan Regulation as a success [4]. And with an estimated 5,000-7,000 rare diseases, only a proportion of which might be susceptible to a drug treatment, the unmet medical need is far from over. It is estimated that just 1% of rare diseases are currently covered by approved treatments in the EU. Therefore, the authors agree with the view that, “*the incentives of the orphan drug legislation are necessary and are responsible for facilitating the development of elegant and sophisticated treatments for patients with real unmet needs*[5].”

Success does not come without a price, however. Many of these so-called “orphan drugs” have higher price points than treatments for more common diseases. To date, governments have, in many cases, taken a more lenient view towards orphan drugs than they have for treatments for more common diseases [6] and, in addition, granted them or their developers special treatment, such as national tax grants; or exempting them from “across the board” price cuts or taxes [7], or other procedures [8]. Such support for orphan drugs and their availability is foreseen in Article 9 of the EU Orphan Regulation, which calls on Member States to support “*research…development and availability of orphan drugs*” [9].

To date, orphan drugs have only accounted for a small percentage of the overall drug budget – for example, they were estimated to account for just 1.9% of pharmaceutical expenditure in Belgium in 2008 [10], rising to around 2% in 2009 [11]; in France and the Netherlands, the 2004 figure was put at 0.7% and 1% of national drug budgets, respectively [12]. Other sources [13] found that in 2007, orphan drugs accounted for 1.7% of the French drug budget, 2.1% in Germany, 1.0% in the UK, 1.5% in Italy and 2.0% in Spain. The average overall impact in these five countries with the highest drug expenditure in Europe is just 1.7% [13]. At this level, the conclusion was that financing orphan drugs appears to be acceptable to governments, when coupled with the societal choice to treat patients with rare diseases. Another study has concluded that the share of the total pharmaceutical market represented by orphan drugs is predicted to increase from 3.3% in 2010 to a peak of 4.6% in 2016 after which it is expected to level off through 2020, as growth falls into line with that in the wider pharmaceutical market. Therefore, fears that growth in orphan drug expenditure will lead to unsustainable cost escalation may not be justified [14].

Nevertheless, it would also appear that, with increasing numbers of orphan drugs, and other developments in healthcare, e.g., stratified medicines [15], governments remain concerned about the impending increased budget impact. A study has estimated that between eight and twelve new orphan drugs will be approved in Europe per year [16], in contrast to a yearly average of six between 2001 and 2010. It is not easy to predict how accurate this will be in practice, given the challenges at all stages of drug development. Additionally, the first orphan drugs approved in 2001 are coming to the end of their 10-year period of “market exclusivity”, intended to prevent similar drugs for the same therapeutic indication being approved for a set period in the EU. Given the size of the markets for treating rare diseases, coupled with the necessary research and development costs, it remains to be seen if this ending of the incentivising protection period will give rise to any meaningful competition in this space. The possibility remains, however, that the ending of the protection period will cause an entry of competitive products in such therapeutic spaces, giving rise to potential price reductions. Member States can also use this point in time – the ending of the 10-year period of market exclusivity and the consequent removal from the EU Community register – to revisit eligibility for national incentives.

Orphan drugs are under the spotlight, something that is likely to continue as the economic crisis in Europe increasingly takes hold and governments respond with austerity measures that include cuts to healthcare expenditures.

## Business models in the orphan drug space – an analysis

There are several different paths that companies have followed to develop orphan drugs. And, in some cases, the pricing of the resulting drugs has led to the perception that unreasonably high prices are being paid for some, not leaving enough space for truly innovative drugs with high value. This can lead to the situation where orphan drugs are not effectively differentiated.

Formally and informally, governments are looking at how they are going to handle orphan drugs in the future. The question is a crucial one, since the health benefits of an orphan drug can only be realised if patients get access to them.

In recent years [17] there has been increasing criticism of behaviours in the orphan drug field, mainly centring on two perceptions of the system. The first is the high prices of orphan drugs and their resulting inability to meet standard cost-effectiveness thresholds. The second is the construct of the system itself and the ability of companies to “game” the system in order to secure the benefits that accrue from being badged as an orphan drug, which may, in turn, allow them to request a higher price point.

These two perceptions, or a combination of them, has led to an increasing questioning of the value of the orphan system in Europe, or pushed some governments to refuse reimbursement.

The authors hypothesise that by examining these criticisms individually, one might be able to turn these different “behaviours” into criteria. These criteria could then be at the basis of a new system for review and assessment of individual orphan drugs, at the time of pricing and reimbursement.

The authors provide below specific, non-exhaustive examples of “behaviours” that can be grouped into two main categories of dynamics: drug indication dynamics (1–3) and regulatory / market dynamics (4–5).

### Multiple indications as opposed to a single, orphan indication

Imatinib, initially authorised for the treatment of Chronic Myeloid Leukaemia (CML) on 27 August 2001, has been repeatedly criticised for having more than one indication; and its subsequent authorisations for GIST (gastrointestinal stromal tumours), ALL (acute lymphoblastic leukaemia), myelodysplastic / myeloproliferative diseases, chronic eosinophilic leukaemia, and dermatofibrosarcoma protuberans have led to questions (so-called “indication creep”) about the system that allowed it. On the one hand, for each indication, there needs to be a separate clinical development programme and a separate request for, and granting of a European Marketing Authorisation – the new indications do not simply accrue from the original. On the other hand, the substance is already known, so some studies do not need to be repeated. There is an apparent expectation from the payers that, as the size of the market for an individual compound increases, the compound ceases to be truly orphan in its applications and, therefore, an expectation that the orphan incentives might no longer be as applicable as at the time of the first authorised orphan indication [18]. Moreover, as indications increase, economies of scale may come into play. In this respect, the literature has consistently found an inverse relationship between orphan drug prices and the prevalence of rare diseases [18].

The current marketing authorisation pathway appears to oblige companies to follow a sequential approach when the mode of action for an active ingredient proves useful in multiple indications, which might undermine this ability. In the context of other initiatives, such as the IRDiRC [19], the need for regulatory approaches to address “repurposing” might be considered.

### Rare into Common – similar questions being raised

A drug approved as an orphan for a specific indication may go on to seek and be granted a Marketing Authorisation for a non-orphan indication. Such a situation could turn a therapy intended initially for a small, well-defined, orphan population into a blockbuster. For example, Bosentan, an orphan medicine for the treatment of Pulmonary Arterial Hypertension (PAH), may also be effective to treat heart failure [20].

### Authorisation first for a non-orphan indication and then for an orphan indication – common into rare

Sildenafil, initially authorised for erectile dysfunction, has become one of the world’s largest-selling drugs. On 28 October 2005, it received a separate Marketing Authorisation as an orphan drug for pulmonary arterial hypertension, a rare condition. Another example is ibuprofen, which is widely used and which has later been shown to be effective in treating patent ductus arteriosis, a rare condition, for which it has received marketing authorisation as an orphan [21].

### Gaining orphan marketing authorisation of an existing therapy – formulation from “magistral” / hospital formula to approved orphan drug for the same condition

Several rare diseases can be treated by existing compounds and, indeed, may have been so for many years. The orphan legislation allows that a company seeking to create standardised production of pharmaceutical-quality versions of such compounds for treating the same condition may receive orphan designation for their version of the drug being approved. Recently, in the EU, amifampridine was granted a marketing authorisation as a designated orphan drug. The branded product is a slight modification of an unlicensed and low-priced compound that has been available for several decades. The price-point requested for the new product was 50-to 70-fold higher compared to the unlicensed formulation. The move caused a group of physicians to write an open letter [22] to the Prime Minister of the UK. Another example from the USA is 17-alpha hydroxyprogesterone caproate (17P), against preterm labour, a drug that had originally received FDA approval in the 1950s for multiple indications and that came off the market in 2000 because of a manufacturing issue. After a publicly funded 2003 study of 463 women, the drug gained widespread use, even though practitioners had to order it from compounding pharmacies [23]. In 2011, the FDA approved 17P under its orphan drug programme, guaranteeing market exclusivity and the drug has been marketed for more than 100 times the price of the compounded product, a price that the company was forced to review after all the criticism received. In Belgium, similar findings have been documented [24].

### The existence of other treatments for the same condition

Although the term “market exclusivity” in the EU’s Orphan Regulation might give rise to the impression that it is not possible to have more than one treatment for the same orphan indication approved within a given period, this is not the case. EU legislation allows for the prohibition of entry of another “similar” medicinal product for the same indication; however, a non-similar product that offers a significant benefit to patients suffering from the rare disease may indeed seek and be granted a marketing authorisation for the same therapeutic indication as an existing orphan product.

There is a perception of “clustering” of multiple treatments for the same indication. For example, currently in Europe there are five treatments for Pulmonary Arterial Hypertension (PAH), authorised at different times. For certain conditions, more than one product have been approved at the same time, such as rilonacept and canakinumab for Cryopyrin-Associated Periodic Syndromes (CAPS), both of which were granted marketing authorisation on the same day. Indeed, the first two approved orphan drugs in the EU were approved on the same day, for the same indication.

### Summary of criticisms and issues arising

These examples threaten to create a climate of increasing uncertainty in an already uncertain field. This could undermine the willingness of companies to invest in the development of orphan drugs. Additionally, the increased uncertainty around successful pricing and reimbursement decisions creates a challenge for companies that face the prospect – at the end of a lengthy and expensive development process – of discovering that their proposed price-point is unacceptable to the payers in a given or multiple markets, resulting in rejections at the time of reimbursement negotiations. This wastes time and money, but also deprives patients of the treatment that they had been expecting.

The issue does not stop there, however. Subsequent evaluations and re-evaluations following public outcries or media campaigns consume resources and loud, public battles call into question the reputation of all stakeholders: industry, which may be accused of “sharp practices”; patients, who may be accused of demanding unreasonable shares of limited resources and, thereby, depriving others from those same resources; and governments, who are called upon to justify their decisions and, often, their reversals of decisions.

## Ways forward?

The solution to this requires a multi-faceted approach. On the one hand, there needs to be a system that lays out clearly and transparently what could be considered a high unmet medical need. But is it also time to evaluate how much a government and the society that it represents might be willing to pay for a solution to that unmet medical need? The issue is about resource allocation: are governments and societies more willing to pay for economic efficiency – treating those patients that maximise health gain subject to a limited budget – or for allocation efficiency – fairness in society? In order to answer this question, there may need to be formal investigations into public preferences on rare diseases, as mentioned above.

It has been acknowledged that standard methodologies for Health Technology Assessments (HTA) will need to be tailored to take into account the specificities of orphan drugs [25,26] given that the higher price-points claimed by orphan drugs are unlikely to meet current cost-effectiveness thresholds where these are applied. This challenge tends to increase proportionally with the rarity of the disease [27]. In its review of the feasibility of HTA methods for so-called “ultra-orphan” drugs in 2005, the National Institute for Health and Clinical Excellence (NICE), noted that there were several criteria that might warrant the creation of a new system [27] – including prevalence, the nature of the condition in question and duration of treatment – and submitted a proposal for creating a tailored system for appraising orphan and ultra-orphan drugs to the Department of Health in 2006. This triggered AGNSS, the Advisory Group for National Specialised Services in the UK to work on the development of a new system to assess “ultra-orphan” drugs.

Evaluations, therefore, need to take into account a different set of benefits or value than simply a cost-effectiveness threshold. A simple lowering of the price of an orphan drug might not provide the answer either, particularly where the disease is very rare, as it would hamper the company’s willingness to produce the drug and place it on the market. For example, in its evaluation of Enzyme Replacement Therapy (ERT) treatment for Gaucher Disease, NICE found that the standard cost-effectiveness figure would have to be increased 10-fold in order to make that treatment fit the standard thresholds. That would translate into a 90% price decrease, which would most likely make the product commercially unviable [25].

It is becoming clear that, in the field of orphan drugs, new methodologies urgently need to be developed in order to address such situations. The authors, therefore, propose that these considerations could form the basis for developing a system for the pricing and reimbursement review of individual orphan drugs, with increased transparency and predictability for the industry on what societies and their governments value, which, in turn could help them make their investment and pricing decisions before choosing to develop and/or market a product. Such a new system would allow the evaluation of treatments for rare diseases on a multi-criteria basis, while allowing the possibility to distinguish between different orphan drugs.

It would also create a framework that would allow governments to distinguish between different new orphan drugs, and allow them to justify their decisions.

Healthcare systems would be more sustainable and the budget redistributed accordingly: paying for those needed drugs that warrant a higher price, while paying less for others, based on a rational and transparent decision-making process.

## Proposed solution

The authors propose that at the time of pricing and reimbursement, each new orphan drug is evaluated against several criteria, which is believed to also help frame a more structured dialogue between manufacturers and payers, with the involvement of the treating physicians and the patients.

Each individual government would then decide on the weighting attributed to each of the criteria in question, based on what each individual society values most, through a survey of public preferences. For that purpose, the authors propose the idea of a discrete choice experiment, involving members of society, to elicit and quantify the relative important of each criterion in the evaluation of orphan drugs.

In multi-criteria decision analysis, an expert panel defines the relevant decision-making criteria and their relative importance. Each criterion needs to be measurable, so that the degree to which an orphan drug attains the criterion can be assessed. The scores of an orphan drug on the different criteria are aggregated with a view to calculating the overall performance of the orphan drug. Decision-makers then allocate resources based on the ranking of drugs according to their performance scores until the budget is exhausted [28].

The criteria described [Table 1 – with some suggested guidance on the potential parameters – would form part of the system. However, this list of criteria is not exhaustive and there are likely to be other criteria that would need to be factored in, such as quality of life, some of which might even be refined on the basis of future research, such as BURQOL-RD [29].

**Table 1:** – Proposed criteria for evaluation of orphan drugs and corresponding potential parameters

**Criteria**	**Price Differential**		
	**Lower**	**Medium**	**Higher**
Rarity	1:2,000 - 1:20,000 or COMP figures > 3 in 10,000 (11%)	1:20,000 - 1:200,000 or COMP figures 1–3 in 10,000 (51%)	Less than 1:200,000 or COMP figures less than 1 in 10,000 (38%)
Level of research undertaken	Literature review	Building on previous existing knowledge	“Blue-sky” – starting research & development programme in an unknown area
Level of uncertainty of effectiveness	Immature, but promising data	Appropriate surrogate end-points	Robust clinical end-points
Manufacturing complexity	Not complex – small molecule / classic galenic form	Moderately complex	Highly complex biological and galenic form
Follow up measures (additional benefits and associated costs)	Moderate to none	Designed to answer specific, defined, delineated question	Safety and efficacy studies + size and duration of study
Characteristics without direct cost impact			
Disease severity	Morbidity	Mortality / severe invalidity in adulthood	Mortality / severe invalidity as infant
Available alternatives / unmet medical need	Alternatives with similar characteristics	Alternatives – but offering strong innovation to the disease treatment	No alternative
Level of impact on condition / disease modification	Low	Medium	Strong
Use in unique indication or not	Existing orphan or non-orphan indications for the same molecule*	Potential for multiple indications	Unique indication – no other use possible

*N.B. Another element could be the total revenues in the context of multiple indications for the same molecule owned by the same company.

This could be particularly applicable in the case that an orphan designation is granted on the basis of insufficient return on investment clause within the EU legislation [30]. In the case where, e.g., multiple existing indications are owned by another company, the discussion and resulting criteria might not be so straightforward, therefore this system always has to be coupled with an element of dialogue between the various stakeholders.

### How do each of these criteria relate to reimbursement and market access?

#### Rarity

The rarer the disease, the more the research and development of an orphan drug costs [18]. Investment needs to be spread over a smaller number of patients. Clinical trials increase in complexity when there are smaller numbers, globally scattered. For example, where trials involve children, the families might need to travel as well as the patient, which increases the costs. NICE acknowledged that rarity plays a key role, however the authors concur that rarity on its own is unlikely to be enough and that some other criteria need to be taken into account.

#### Level of research undertaken to receive marketing authorisation as an orphan

A company that has conducted literature reviews on the well-proven and established use of an existing compound incurs much less cost to bring the compound to market, compared with those who start from “a blank sheet of paper”, from the natural history of the disease through full-blown clinical development and likely follow-up. Indeed, a company’s commitment to conduct both an extensive clinical development programme and outcomes research programme may be favoured by payers afterwards. Outcomes research studies are crucial to document: the natural history of the disease, the unmet need through patient surveys, the current direct and indirect cost burden – all these elements of knowledge are scarce. The generation – or not – of such new data by a manufacturer could also be an objective criteria for assessment.

#### Level of uncertainty

A company that has conducted a full clinical development programme that includes robust, validated clinical endpoints incurs more costs than those who do not. Products with a high level of uncertainty are risky bets for providing value for money. Uncertainty comprises several sub-parameters, which are important elements in comparing one technology with another. Elements include whether clinical trials are randomised or not, whether an active comparator has been used – or at least the standard of care – versus placebo-controlled trials, the size of the patient population studied and the level of statistical significance of the treatment effect. Individual agencies that may be willing to accept lower requirements for quantitative data are less willing to do so for the qualitative basis of evidence [31].

#### Manufacturing complexity

Different pharmaceutical technologies have a different cost to produce even after Marketing Authorisation. The requirements from a regulatory perspective (including safety, quality, efficacy, risk-management) vary between a simple chemical compound and a complex biological, and between different technologies (e.g., emerging technologies based on human tissue and gene therapy, as opposed to classical oral forms). The manufacturing risks that a company must bear – inherent to the technology in question – are also different, therefore also incurring different costs.

#### Follow-up measures

The higher the uncertainty at the time of Marketing Authorisation, the more likely a treatment is to require follow-up measures, regulatory or otherwise. These can vary in size, duration, objective, complexity and, therefore, costs. This is likely to be further exacerbated as a function of increasing rarity.

#### Disease severity

Severe diseases have a big impact on society at large and families in particular. Hospitalisations, symptomatic care, disability, absence from work and exemption from tax contributions, amongst others, are a societal burden that national budgets have to bear. NICE citizens’ council mentioned that the society would be willing to pay more for a disease that is rare and very serious. A collaborative research project is current being undertaken at EU level to attempt to capture and quantify these costs [29]. This may give further input for consideration in the development of any system.

#### Available alternatives as opposed to unmet medical need

It is clearly a societal, ethical and moral value to treat someone who has no medical alternative. European governments did take a decision that “*patients suffering from rare conditions should be entitled to the same quality of treatment as other patients*” [1]. Furthermore, entering a field where there is no existing treatment is a different proposition – particularly in the rare disease one. There are additional costs and activities inherent in being the first treatment to come to the market. Disease awareness is likely to be very low and, in order to make a serious impact on the disease treatment, it may be necessary to embark on a series of costly disease-awareness programmes.

#### Level of impact on condition / disease modification

A drug that is truly disease-modifying or transformative will inherently be more valuable in terms of outcome than a treatment with a moderate impact, e.g., palliative, symptomatic, disease-stabilising or substitutive. The ultimate example of this could be a true “cure” which would remove the need for all other medical interventions and, therefore, expenses to the healthcare systems.

#### Use in unique indication or not

A drug that only treats one condition and will not have a use in any other – e.g., some of the ERTs for use in Lysosomal Storage Disorders – has a very different potential business prognosis than a molecule that either is, or can be, used in a wider population. Companies that receive Marketing Authorisation for several indications of the same molecule accrue higher revenues than those whose molecule is only applicable in one indication [32]. The first could, therefore, share its benefits with society and, in particular, with governments; while, for the latter, governments could be sharing the burden in order to still allow such companies a fair return on investment and therefore help sustaining research and innovation.

## Conclusion

Orphan drugs and rare diseases are a field where little is known. Even after a drug has been on the market for many years, information may remain patchy and scattered. Knowledge is growing all the time and the only way forward is a collaborative one, which is one of the hallmarks of the rare disease space.

Orphan drugs are defined under EU law on the basis of medical need, amongst other criteria. However, several payers believe that this definition is too broad in its application and wish to see a more focussed approach. It is clear that not all orphan drugs display the same characteristics. As governments seek to understand the value of an individual orphan drug – in all its impacts – from the value to the patient itself, to the value to society in granting more active participation, in the economic investment in R&D, in the value that an individual orphan drug might give to increasing knowledge elsewhere [33], there is a need to develop a system that not only is adapted to the specificities of orphan drugs, but also one that provides clear and transparent guidance in the decision-making process.

This is not something that can be addressed by the European legislation, which does not and cannot cover pricing and reimbursement, which remains a Member State competence. Pricing a product is beyond the designation, development and marketing authorisation processes contained in the EU Regulation. Additionally, there appears to be no dispute about the fact that patients suffering from a rare disease deserve the same level of proven safety, quality and efficacy in their medicines as patients suffering from more common diseases [1].

On the other hand, in recent years, a series of business models in the field of orphan drugs have attracted criticism for various reasons. While analysing what could be considered “abuse” of the system when it comes to pricing and reimbursement, one might actually find out the keys to a solution – the opposite of the implied abuse also implies that the alternative behaviour is something that governments and/or society would value.

The authors propose the development of a new assessment system based on several weighted evaluation criteria [Table 1], which would serve as a tool for Member State governments, allowing them to value an orphan drug that fulfilled all the criteria very differently from one that only met some of them. An individual country could determine the (monetary) value that it places on each of the different criteria, according to societal preferences, the national healthcare system and the resources at its disposal. A new system could, however, offer pricing and reimbursement decision-makers a tool to handle the different characteristics amongst new orphan drugs, and to redistribute the national budgets in accordance with the outcome of a differentiated assessment.

## Competing interests

The authors declare that they have no competing interests.

## Authors’ contribution

WHW, AP, AS & SS have conceived the initial concept. WHW and AP have investigated the potential structure and drafted the first proposed manuscript. AS and SS have further complemented the initial drafts with referential data and expert contributed input. All authors read and approved the final manuscript.
